# Characteristics and outcomes of diabetic patients with an implantable cardioverter defibrillator in a real world setting: results from the Israeli ICD registry

**DOI:** 10.1186/s12933-016-0478-2

**Published:** 2016-12-01

**Authors:** Hillel Steiner, Michael Geist, Ilan Goldenberg, Mahmoud Suleiman, Michael Glikson, Alexander Tenenbaum, Moshe Swissa, Enrique Z. Fisman, Gregory Golovchiner, Boris Strasberg, Alon Barsheshet, On behalf of the Israeli Working Group of Pacing and EP

**Affiliations:** 1 The Edith Wolfson Medical Center, Holon, affiliated with the Sackler School of Medicine, Tel Aviv University, Tel Aviv, Israel; 2 The Chaim Sheba Medical Center, Tel Hashomer, affiliated with the Sackler School of Medicine, Tel Aviv University, Tel Aviv, Israel; 3 Rambam Medical Center, Haifa, Israel; 4 Cardiovascular Diabetology Research Foundation, Holon, Israel; 5 Kaplan Medical Center, Rehovot The Hebrew University, Jerusalem, Israel; 6 Rabin Medical Center, Petah Tikva, Israel; 7 Department of Cardiology, The Edith Wolfson Medical Center, Holon, Israel

**Keywords:** Implantable cardioverter defibrillator, Diabetes mellitus, Heart failure, Outcomes

## Abstract

**Aims:**

There are limited data regarding the effect of diabetes mellitus (DM) on the risks of both appropriate and inappropriate implantable cardioverter defibrillator (ICD) therapy. The present study was designed to compare the outcome of appropriate and inappropriate ICD therapy in patients with or without DM.

**Methods and results:**

The risk of a first appropriate ICD therapy for ventricular tachyarrhythmias (including anti tachycardia pacing and shock) was compared between 764 DM and 1346 non-DM patients enrolled in the national Israeli ICD registry. We also compared the risks of inappropriate ICD therapy, and death or cardiac hospitalization between diabetic and non-diabetic patients. Diabetic patients were older, were more likely to have ischemic cardiomyopathy, lower ejection fraction, atrial fibrillation, and other co-morbidities. The 3-year cumulative incidence of appropriate ICD therapy was similar in the DM and non-DM groups (12 and 13%, respectively, p = 0.983). Multivariate analysis showed that DM did not affect the risk of appropriate ICD therapy (HR = 1.07, 95% CI 0.78–1.47, p = 0.694) or inappropriate therapy (HR = 0.72, 95% CI 0.42–1.23, p = 0.232). However, DM was associated with a 31% increased risk for death or cardiac hospitalization (p = 0.005). Results were similar in subgroup analyses including ICD and defibrillators with cardiac resynchronization therapy function recipients, primary or secondary prevention indication for an ICD.

**Conclusions:**

Despite a significant excess of cardiac hospitalizations and mortality in the diabetic population, there was no difference in the rate of ICD treatments, suggesting that the outcome difference is not related to arrhythmias.

## Background

Several randomized trials have shown that an implantable cardioverter–defibrillator (ICD) can improve survival both among patients who have had sustained ventricular tachyarrhythmias and among selected patients who have systolic heart failure (HF) without ventricular arrhythmia [1–3]. Patients with evidence of systolic HF with intraventricular conduction delay may further benefit by implantation of a defibrillator with cardiac resynchronization function (CRTD) which may improve left ventricular function, prevent heart failure events and survival [4, 5] while obesity in mild heart failure did not diminish the clinical benefit of cardiac resynchronization therapy to reduce risk for appropriate ICD therapy [6].

Patients with diabetes mellitus (DM) are at increased risk for sudden cardiac death and heart failure [7–14]; a previous study suggests that diabetes mellitus may affect appropriate and inappropriate ICD discharge [14]. In addition, patients with obesity and overweight derived more benefit from CRT. Higher BMI was independently associated with better clinical outcome in CRT patients [15].

Importantly, little is known about the rates of ICD and CRTD therapies in DM patients as compared to non DM patients in a real life setting. Thus, we aimed to investigate whether DM may affect appropriate ICD therapy, inappropriate ICD therapy, HF hospitalization or death among patients enrolled in the Israel ICD registry.

## Methods

### Study population

The Israeli ICD Database is a prospective, national, multi-center registry of all patients implanted with an ICD or CRT-D for primary and secondary prevention in the 21 implanting centers of Israel [16, 17]. The registry was initiated in July, 2010, and prospective follow up was started in July, 2011. At baseline, clinical and implantation characteristics were entered by the local electrophysiologist into a secure, web based electronic case report form. Follow up data for clinical and arrhythmic events were obtained from consecutively enrolled patients at 6 month intervals.

Patients were classified as having DM if they reported treatment for DM at the index hospitalization for device implantation or replacement or they were diagnosed with DM according to their medical chart. Other clinical variables collected included basic demographics, indication for implantation, electrocardiographic QRS morphology, left ventricular ejection fraction, New York Heart Association (NYHA) functional class, co-morbidities, and medications. The registry was approved by the institutional review board of each participating center and patients were included after providing written informed consent.

### Endpoints

The endpoints evaluated at follow up included all-cause mortality and hospitalization for heart failure as well as device therapies that were further classified as being appropriate or inappropriate therapies including antitachycardia pacing (ATP) and ICD shock. All intra-cardiac electrograms of therapies were reviewed by the attending electrophysiologist who determined if the therapies were appropriate or not.

### Statistical methods

Continuous variables were expressed as mean ± standard deviation (SD). Categorical data were summarized as frequencies and percentages. Characteristics of patients categorized by diabetes status were compared by the Wilcoxon rank-sum test or Chi square test, as appropriate. The probabilities of appropriate ICD therapy, inappropriate ICD therapy and cardiac hospitalization or death by DM status were graphically displayed according to the method of Kaplan and Meier, with comparison of cumulative events by the log-rank test.

The best subsets regression procedure was used to identify significant variables to be included in the multivariate regression models. For a model with the endpoint of death or cardiac hospitalization the variables included age, sex, ischemic heart disease, history of atrial fibrillation, NYHA functional class III–IV vs. I–II, creatinine level, and DM that was forced into the model. For a model with appropriate ICD therapy endpoint the variables included age, primary vs. secondary prevention indication, history of atrial fibrillation, QRS width, history of any ventricular arrhythmias (non-sustained or sustained VT), LVEF, device type (CRTD vs. ICD), and DM which was forced into the model. Analyses were conducted with SAS software (version 9.4, SAS institute, Cary, NC, USA). A 2-sided p value <0.05 was used for declaring statistical significance.

## Results

From July 2010 through December 2014 a total of 2110 patients underwent ICD or CRTD implantations in 21 centers in Israel for whom follow up data was available. Patients were followed for a mean ± SD of 21 ± 10.2 months.

We identified 764 (36%) patients with DM and 1346 (64%) without DM. The DM patients were significantly more ill in a wide variety of ways: they tended to be older, and had more co-morbid conditions, including atrial fibrillation, chronic lung disease, end stage renal failure on dialysis, hypertension, cerebrovascular disease, smoking and sleep apnea (Table 1). Diabetics were more likely to have more severe congestive HF, a wider QRS, a lower ejection fraction, to undergo CRTD implantation and to have ischemic cardiomyopathy.

**Table 1:** Patient characteristics

	Non-diabetics (%)	Diabetic (%)	p value
N	1346	764	
Male	1215 (82)	726 (85)	0.033
Age (mean ± SD)	62.2 ± 14	66.3 ± 9.4	<0.001
Age ≥75	256 (19)	163 (21)	0.154
Prior VA	452 (34)	273 (31)	0.252
Primary prevention	901 (66)	589 (76)	<0.001
Ischemic heart disease	904 (67)	625 (82)	<0.001
History of AF	254 (19)	170 (22)	<0.001
Chronic lung disease	85 (6)	118 (16)	<0.001
Smoker	379 (29)	272 (36)	0.003
Dialysis	12 (1)	23 (4)	<0.001
Sleep apnea	81 (6)	89 (12)	<0.001
Prior CVA	98 (7)	67 (9)	<0.001
Atrial fibrillation	30 (2)	35 (1)	0.05
NYHA ≥3	377 (28)	326 (43)	<0.001
EF	30.5 ± 11.6	28.0 ± 8.3	<0.001
QRS duration	115.8 ± 29.8	124.6 ± 30.9	<0.01
CRTD	412 (31)	332 (43)	<0.001
ACE inhibitor	921 (69)	603 (79)	<0.001
Beta Blocker	1044 (78)	666 (87)	<0.001

*VA* ventricular arrhythmia, *CVA* cerebrovascular accident, *AF* atrial fibrillation, *NYHA* New York Heart Association, *EF* ejection fraction, *CRTD* cardiac resynchronization therapy-defibrillator, *ACE* angiotensin converter enzyme

DM patients implanted with a defibrillator (ICD or CRTD) had poor prognosis compared with non- diabetic patients implanted with a defibrillator.

**Fig. 1:**
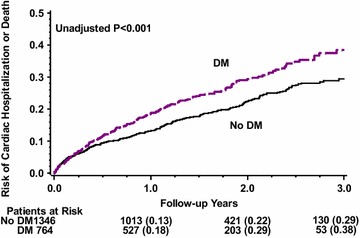
Cumulative probability of cardiac hospitalization or death among diabetic and non-diabetic patients

At 3 years of follow up, the cumulative event rate of cardiac hospitalization or death was 38% among DM patients and 29% among no-DM patients (p < 0.001, Fig. 1). The cumulative event rate of death was 12 and 6% in DM and no-DM patients, respectively (p = 0.021).

**Fig. 2:**
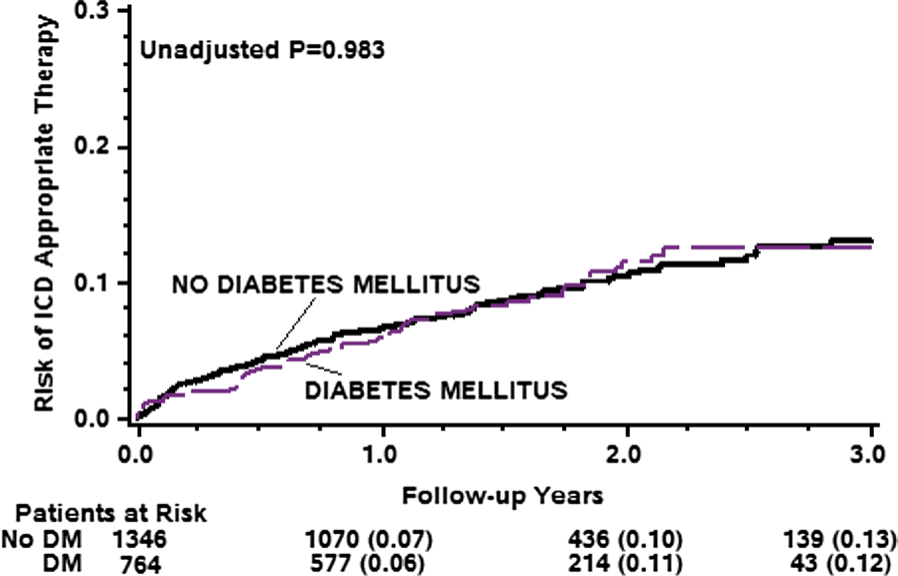
Cumulative probability of appropriate ICD therapy among diabetic and non-diabetic patients

**Fig. 3:**
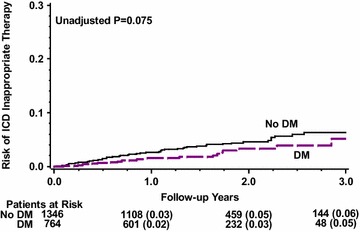
Cumulative probability of inappropriate ICD therapy among diabetic and non-diabetic patients

By multivariate analysis (Table 2), DM was associated with a significant 31% increased risk for death or cardiac hospitalization (p = 0.005), and a trend towards 1.5-fold increased risk for death (p = 0.104). During study follow up 114 patients (7%) with a primary prevention indication and 82 patients (16%) with a secondary prevention indication received an appropriate ICD therapy. There were also 78 patients (4%) who experienced an inappropriate ICD therapy during study follow up. However, there was no significant difference in the incidence of appropriate ICD therapy between the groups (Fig. 2). At 3 years of follow up the cumulative event rate of appropriate ICD therapy was 12% in the DM patients and 13% in the no-DM patients (p = 0.983). Similarly, there was no significant difference in the incidence of inappropriate ICD therapy between the groups (Fig. 3) with 5% among the DM patients and 6% among the non-DM patients (p = 0.075, Fig. 2).

**Table 2:** Multivariate analysis: diabetes mellitus and the risk of cardiac hospitalization or death

	Hazard ratio	95% CI	p value
Cardiac hospitalization or death	1.31	1.08–1.57	0.005
Death	1.49	0.92–2.41	0.104
Cardiac hospitalization	1.23	1.00–1.50	0.047

Adjusted for age, sex, ischemic heart disease, history of atrial fibrillation, NYHA functional class III–IV vs. I–II, and creatinine level

Consistent with these findings, multivariate analysis (Table 3) showed that DM did not affect the risk of appropriate ICD therapy [hazard ratio (HR) = 1.07, p = 0.694], including appropriate shock (HR = 1.13, p = 0.519) and appropriate ATP (HR = 1.07, p = 0.743). DM also did not affect the risk of inappropriate ICD therapy (HR = 0.72, p = 0.232) including inappropriate shock (HR = 0.67, p = 0.312), and inappropriate ATP (0.82, p = 0.517, Table 3).

**Table 3:** Multivariate analysis: diabetes mellitus and the risk of appropriate and inappropriate ICD therapies

	Hazard ratio	95% CI	p value
Appropriate therapies*	1.07	0.78–1.47	0.694
Appropriate ATP	1.13	0.79–1.62	0.519
Appropriate shock	1.07	0.70–1.64	0.743
Inappropriate therapies^#^	0.72	0.42–1.23	0.232
Inappropriate ATP	0.67	0.31–1.46	0.312
Inappropriate shock	0.82	0.44–1.51	0.517

* Adjusted for age, primary vs. secondary prevention indication, history of atrial fibrillation, QRS width, history of any ventricular arrhythmias (non-sustained or sustained VT), LVEF, and CRTD

^#^ Adjusted for age, sex, primary vs. secondary prevention indication, history of atrial fibrillation, LVEF, NYHA functional class III–IV vs. I–II, and CRTD

Results were similar in subgroup analyses, demonstrating that DM was not associated with a significant increased risk for an appropriate therapy among ICD recipients (HR = 1.04, p = 0.843) or among CRT-D recipients (HR = 1.14, p = 0.636), among patients with a primary prevention (HR = 1.00, p = 0.985) or among patients with a secondary prevention indication for an ICD (HR = 1.15, p = 0.572).

## Discussion

The relationship between glucose metabolism abnormalities and atrial fibrillation [18–20] as well as several other types of rhythm disturbances is well established in both clinical and experimental models [21–24]. In this context, our findings have several important implications regarding the risk associated with DM in a “real world” setting among patients implanted with a defibrillator (ICD or CRTD). We have shown that (1) DM patients implanted with a defibrillator have a significantly higher risk for cardiovascular hospitalization or death compared with no-DM patients. (2) Despite the poorer prognosis among DM patients, there was no significant difference in the risk for appropriate or inappropriate ICD therapies compared with no-DM patients.

Overall, the DM patient group had a higher risk profile for poor outcomes, being older, having more comorbidities specifically pulmonary disease and renal failure, and more advanced heart failure. Thus it could be anticipated that they would have a worse prognosis. This finding is consistent with other studies of ICD patients who have found DM to be an independent risk factor for mortality [25–27].

In our study, DM was not associated with an increased rate of ICD therapies of any type- including appropriate or inappropriate shocks or anti tachycardia pacing. This is in concordance with the post hoc sub group analyses of the comparison of medical therapy, pacing, and defibrillation in heart failure (COMPANION) and multicenter automatic defibrillator implantation trial (MADIT)II trials that did not find an increased rate of appropriate therapies in the DM cohort [28, 29]. However, a recent sub-study of the multicenter automatic defibrillator implantation trial-reduce inappropriate therapy (MADIT RIT) did find that DM patients had a 58% increased risk of appropriate therapy (p = 0.003), but a 46% decreased risk of inappropriate therapy (p = 0.002) compared to no DM patients during a mean follow up of 17 months [14]. The authors suggested that the increased risk for appropriate ICD therapy might be explained by a reduced autonomic function or vulnerable myocardium resulting from ischemia and fibrosis in DM patients rendering the myocardium more prone to produce ventricular arrhythmias. The authors hypothesized that the lower risk of inappropriate therapy in DM patients observed in their study might be explained by a lower likelihood of DM patients experiencing exercise-induced sinus tachycardia or rapid ventricular response due to the fact that patients with diabetes mellitus may be more immobile and sedentary, and may have autonomic dysfunction or neuropathy.

The discrepancies between our findings and the MADIT RIT sub study, a primary prevention trial, may result from a different patient mix. Our registry included much more ischemic cardiomyopathy patients in both the diabetics and non-diabetics subgroups (87/67%) than MADIT RIT (64/48%). Our registry did include 30% implanted for secondary prevention—with far more non-diabetics in the high arrhythmia risk secondary prevention group (Table 1). However, we performed a subgroup analysis showing that DM was not associated with a significant increased risk for an appropriate or inappropriate therapy both among patients with a primary prevention or among patients with a secondary prevention indication for an ICD.

Our findings indicate that although DM patients do have a poor prognosis, the excess in mortality or hospitalization for heart failure is not due to ventricular tachy-arrhythmias. DM causes other forms of heart failure not amenable to device therapy such as heart failure with a normal ejection fraction, thus the ICD may not prevent mortality from this form of heart failure. Some studies have found DM to be a risk factor for sudden cardiac arrest [8–11, 30] which is predominantly due to ventricular arrhythmias [31]. DM was thought to cause repolarization abnormalities, predisposing to these arrhythmias [12]. This assumption needs reconsideration.

Our findings show that attempts to reduce the excess cardiac mortality in DM patients should not focus on the treatment of arrhythmia, but on other aspects of diabetic treatment. Although glycemic control in the past did not improve cardiovascular outcomes of diabetic patients [32], newer treatment modalities have been promising. Recent studies have shown that standard treatment of heart failure may be augmented by inhibitors of sodium-glucose cotransporter 2 and glucagon-like peptide 1 analogues, both of whom may reduce cardiac mortality of DM patients in randomized, controlled trials [33–35].

Our other finding was that there was no difference in the rate of inappropriate shocks between the two groups. DM is a risk factor for development of atrial fibrillation [7, 13, 36–38] and indeed, more of our patients had atrial fibrillation (Table 1), a major cause of inappropriate shocks, and it would be anticipated that the rate of inappropriate shocks would be increased. Evidence based programming of these devices has greatly decreased the rate of inappropriate therapies especially in diabetics [14] and indeed several centers contributing to the registry were participants in successful programming trials during the course of follow up, which may explain why there was no difference in the rate of inappropriate therapies [14, 39].

There other possible explanations for our findings. Current medical regimens have effectively reduced the rate of ICD therapies to the point that ICD implantation in non-ischemic cardiomyopathy is not beneficial, and this may be true in ischemic cardiomyopathy as well. However, a significant difference did remain in the other cardiovascular outcomes.

A limitation of our study is the designation of patients as diabetics based on self-reporting rather than laboratory results such as serum glucose or glycosylated hemoglobin (HbA1c) levels that constitute the criteria for the diagnosis of diabetes. Missing also is data regarding the length of time since diagnosis and the degree of glycemic control and non-cardiac organ failure. Some patients classified as non-diabetics at entry may have developed diabetes during the follow up period, however the study mean follow up of 22 months would not be expected to cause target organ damage.

In summary, based on the data from the Israeli ICD registry we conclude that DM patients implanted with ICDs have increased mortality and hospitalizations as compared to no DM patients, but a similar rate of appropriate and inappropriate therapies. Further attempts to reduce the excess mortality and morbidity associated with DM in heart disease should target other aspects of heart failure in this high risk population.

## Abbreviations

ATP: anti-tachycardia pacing
CRTD: cardioverter-defibrillator with cardiac resynchronization therapy
COMPANION: comparison of medical therapy, pacing, and defibrillation in heart failure
DM: diabetes mellitus
HF: heart failure
ICD: implantable cardioverter-defibrillator
MADIT: multicenter automatic defibrillator implantation trial
MADIT RIT: multicenter automatic defibrillator implantation trial-reduce inappropriate therapy trial
NYHA: New York Heart Association
SD: standard deviation
